# Isolation and characterization of WUPyV in polarized human airway epithelial cells

**DOI:** 10.1186/s12879-020-05224-y

**Published:** 2020-07-09

**Authors:** Chao Wang, Tianli Wei, Yiman Huang, Qiong Guo, Zhiping Xie, Jingdong Song, Aijun Chen, Lishu Zheng

**Affiliations:** 1 NHC Key Laboratory of Medical Virology and Viral Diseases, National Institute for Viral Disease Control and Prevention, China CDC, 100 Ying-Xin St., Xi-Cheng District, Beijing, 100052 China; 2 Department of Pediatrics, Beijing Friendship Hospital, Capital Medical University, 95 Yong-An St., Xi-Cheng District, Beijing, 100050 China

**Keywords:** WU polyomavirus, Human polyomavirus, Human airway epithelial cell, Virus isolation, Respiratory infection

## Abstract

**Background:**

Washington University polyomavirus (WUPyV) is a novel human polyomavirus detected in childwith acute respiratory infection in 2007. However, the relationship between WUPyV and respiratory diseases has yet to be established for lacking of a suitable in vitro culture system.

**Methods:**

To isolate WUPyV with human airway epithelial (HAE) cells, the positive samples were incubated in HAE, and then the nucleic acid, VP1 protein and virions were detected using real-time PCR, immunofluorescence and electron microscopy respectively.

**Results:**

The result showed that WUPyV could replicate effectively in HAE cells and virions with typical polyomavirus characteristics could be observed. Additionally, the entire genome sequence of the isolated strain (BJ0771) was obtained and phylogenetic analysis indicated that BJ0771 belongs to gene cluster I.

**Conclusions:**

Our findings demonstrated clinical WUPyV strain was successfully isolated for the first time in the world and this will help unravel the etiology and pathogenic mechanisms of WUPyV in respiratory infection diseases.

## Background

Washington University polyomavirus (WUPyV) is a small, nonenveloped virus, containing a circular double-stranded DNA genome that belongs to the genus *Betapolyomavirus* in the *Polyomaviridae* family. It was first identified by Gaynor et al. in 2007 from a child with pneumonia using high-throughput sequencing [1, 2]. The International Committee on Taxonomy of Viruses (ICTV) named it human polyomavirus 4 (HPyV 4).

WUPyV now has a worldwide distribution and the detection rate in children with acute respiratory tract infections (ARTIs) ranges from 0.4 to 16.4% [2]. The main clinical characteristics of infection are tachypnea, hypoxia, fever, cough, wheezing, expectoration, and a runny nose, some patients have also reported vomiting and diarrhea [3, 4]. In addition to respiratory secretions, WUPyV can also be detected in blood, urine, feces, and cerebrospinal fluid samples [5, 6]. Nucleic acid and serological studies showed that WUPyV positive rates among the general population were 6.7 and 80%, respectively [7, 8], indicating that WUPyV infection is widespread in the human population. However, the relationship between WUPyV and respiratory disease is still unclear, because WUPyV is frequently co-detected with other respiratory viruses [9, 10], and a lack of cell culture system has limited research on the infectivity and pathogenicity of WUPyV [11, 12].

Human airway epithelial (HAE) cells are the primary target for many respiratory viruses in vivo. Compared with traditional cell lines, HAE cells reconstituted in vitro can provide an appropriate cellular environment and have been widely used in research about the etiology, pathogenic mechanisms, and potential treatment methods of respiratory viruses, including influenza viruses, parainfluenza virus, Nipah virus, bocavirus, and respiratory syncytial virus [13–17]. The air–liquid interface (ALI) is a commonly used technique for culturing HAE cells, first established by Whitcutt et al. [18]. ALI-HAE cells can reconstruct a pseudostratified architecture composed of basal, ciliated, mucus-producing goblet cells, and its defense mechanisms [19]. Recently, the new coronavirus (SARS-CoV-2) causing an outbreak in Wuhan, China, was isolated using ALI-HAE cells, providing strong support for the identification of pathogens during the epidemic [20].

In this study, we successfully isolated a WUPyV strain (BJ0771) from a clinical sample for the first time in the world using ALI-HAE cells. The identity of the virus particles was confirmed by immunofluorescence and electron microscopy. We further explored the replication characteristics of WUPyV and obtained its whole genome sequence.

## Methods

### Specimen collection and detection

Nasopharyngeal aspirates (NPAs) of hospitalized children with ARTI (≤14 years) were collected from the Beijing Friendship Hospital (China). WUPyV-VP1 gene fragments were detected by real-time PCR using the TaqMan Gene Expression Master Mix (Thermo Fisher Scientific, USA) according to the manufacturer’s protocol. The following primers were used: forward primer: 5′-CCAATGGTACTGTGCCTCATGT-3′, reverse primer: 5′-CCATGATTCAATGCTGTACTTTTCA-3′, and probe: FAM-ATTCCAGTTCTGAAACACCCAGGGCAAG-TAMRA. The PCR products were confirmed by sequencing.

### ALI-HAE cell culture

Primary HAE cells were kept in our laboratory. ALI-HAE cells were cultured according to methods previously established in our laboratory [21]. Briefly, HAE cells were cultured on a type I/III collagen-covered six-well plate with BEGM medium (Lonza, Germany) containing additives in a 37 °C, 5% CO_2_ incubator. Once the cells reached 80–90% confluence, they were digested with trypsin and plated at a density of 3 × 10^5^ cells/well on type IV collagen-coated Transwell plates (Costar, USA), and the culture medium for both the apical and basolateral sides were renewed every other day. Once the cells formed tight junctions, the medium was replaced with HAE 3D medium (BEGM + DMEM + additives) and cultured for 5 days, then HAE cells were cultured at an ALI for 4–6 weeks until the cells were well differentiated into a 3D pseudostratified structure.

### Clinical virus isolation on HAE cells

One of WUPyV-positive samples with a Ct value less than 20 (BJ0771, Ct = 12.47) was selected for virus isolation. The positive samples were added to the apical layer at a density of 100 copies/cell (calculated based on the number of cells initially added to the Transwell insert). After 2 h of incubation, HAE cells were rinsed twice with PBS, and maintained in a 37 °C, 5% CO_2_ incubator. Then, 200 μL of medium was added to the apical layer following a 2 h incubation at specific time points post-infection for harvesting the virus. Nucleic acids were extracted from both the apical washes and the basal medium for real-time PCR, and then a replication curve was constructed.

### Immunofluorescence analysis

Rabbit antiserum against WUPyV VP1 developed in our laboratory was used in immunofluorescence analysis. The WUPyV VP1 gene (GenBank accession no. ABQ09289) was ligated to the pET-30a vector (Novagen, USA) and the recombinant histidine-tagged VP1 was expressed in BL21 *E. coli*. The protein was then purified through a Ni column (QIAGEN, Germany) and used to immunize rabbits twice, and finally the serum was obtained. HAE cells were infected with harvested WUPyV at 100 copies/cell, then fixed with 4% paraformaldehyde for 30 min at 14 days post infection (dpi) and permeabilized with 0.2% Triton X-100 for 2 h. After blocking with 5% bovine serum albumin (BSA) for 1 h, cells were incubated with primary antibodies diluted in 1% BSA overnight at 4 °C (WUPyV-VP1 antiserum, 1:3000, *β*-tublin, 1:100, Sigma, USA). Then cells were incubated with secondary antibodies at 37 °C for 2 h (goat-derived Dylight 488-labeled anti-mouse IgG and goat-derived Dylight 594-labeled anti-rabbit IgG, 1:500, Thermo Fisher Scientific, USA). Nuclei were counterstained with DAPI (Sigma, USA). Confocal images were recorded using a confocal microscope (Zeiss LSM880, Germany).

### Transmission electron microscopy

HAE cells were incubated with harvested WUPyV at 100 copies/cell and the supernatants were collected when the viral genome copy number reached 10^9^ copies/μL. The supernatants were then observed by transmission electron microscopy (FEI TF20, USA).

### WUPyV genome sequencing

For complete genome sequencing of WUPyV, six pairs of primers containing overlap were designed according to the WUPyV reference strain B0 (GenBank accession number: EF444549), as shown in Table 1. Ex Taq (TaKaRa, China) was used to perform PCR amplification of the isolated WUPyV strain. The PCR products were sequenced by Beijing Tsingke Biological Technology, and the sequences were then assembled using Sequencher 5.0 software to obtain the whole genome sequence. A maximum likelihood phylogenetic tree was generated using MEGA 7.0 software, and homology of the nucleotide and amino acid sequences was analyzed using BioEdit.

**Table 1:** Primer sets for amplification of the whole genome of WUPyV

Primers	Primer sequence (5′-3′)	Location (nt)^a^	Annealing temp(°C)
A-(F)	GCCTCAGGCCTCCTTATT	1–18	51
A-(R)	GAAGGGTAGAAGCATCATAAAC	14,621,483	
B-(F)	AATGGCACTGGCACCTATCC	1002–1021	52
B-(R)	CTCCAACCATTCTGCCAAAG	2338–2357	
C-(F)	TTTTGGGCAGTTGGAGGAC	2123–2141	51
C-(R)	GTGCTGCTTTACTTGACCTTTGT	3379–3401	
D-(F)	AGGAGCCAAAGTAGCAGGGAC	3091–3111	54
D-(R)	TGTTCACAGGCAACACCACC	4337–4356	
E-(F)	CAGCACTAACTCTATGTCTAAAAGG	4086–4110	51
E-(R)	TTTCTGAACTAAAAGCAGAGGG	5193–5214	
X-(F)	TTTGCTTGTGGCGCTTGT	4731–4748	49
X-(R)	TTTCAGGCACAGCAAGCAAT	582–601	

^a^ The locations are based on the reference genome sequence of strain EF444549

## Results

### Clinical information about the patient infected with WUPyV

From Apr. 2017 to Mar. 2018, 76 (5.96%) WUPyV positive samples were detected among 1276 hospitalized children. WUPyV positive samples with a Ct value less than 20 were selected to infect HAE, and a strain of WUPyV (BJ0771) was isolated from a 3-year-old child who was diagnosed with asthmatic bronchopneumonia in Beijing Friendship Hospital, China. The patient was hospitalized in November, 2017, with symptoms of fever, cough, wheezing, runny nose and vomiting.

### WUPyV replication kinetics in HAE cells

**Fig. 1:**
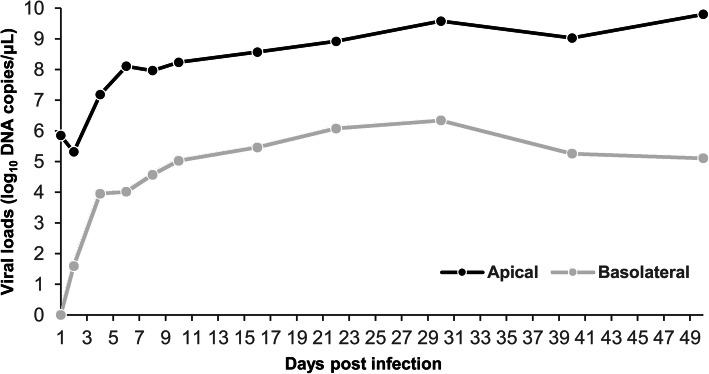
Replication kinetics of the WUPyV clinical isolate in HAE cells. HAE cells were inoculated with diluted nasal aspirates (100 copies/cell). Black points represent viral loads in the apical washes, and orange points represent viral loads in the basolateral medium. Data are presented as WUPyV log10 DNA copies/μL

To determine whether HAE cells could be used to propagate WUPyV, 200 μL of medium were collected from both the apical and basolateral chambers throughout the course of infection, then viral DNA was extracted and tested by real-time PCR. The replication curves showed that WUPyV infection was long lasting, the viral loads increased rapidly from 2 to 6 days post infection (dpi), with a nearly 400-fold increase in virus particles in the apical washes, and finally reached a plateau around 10 dpi and then maintained a level of about 10^9^ copies/μL. Additionally, the viral genome was detected in the basolateral medium at 2 dpi, and the viral load increased gradually with time until 30 dpi. The viral loads in the basolateral medium were ~ 3 log less than the copy numbers released from the supernatant over the course of infection (Fig. 1).

### Immunofluorescence of WUPyV in HAE cells

**Fig. 2:**
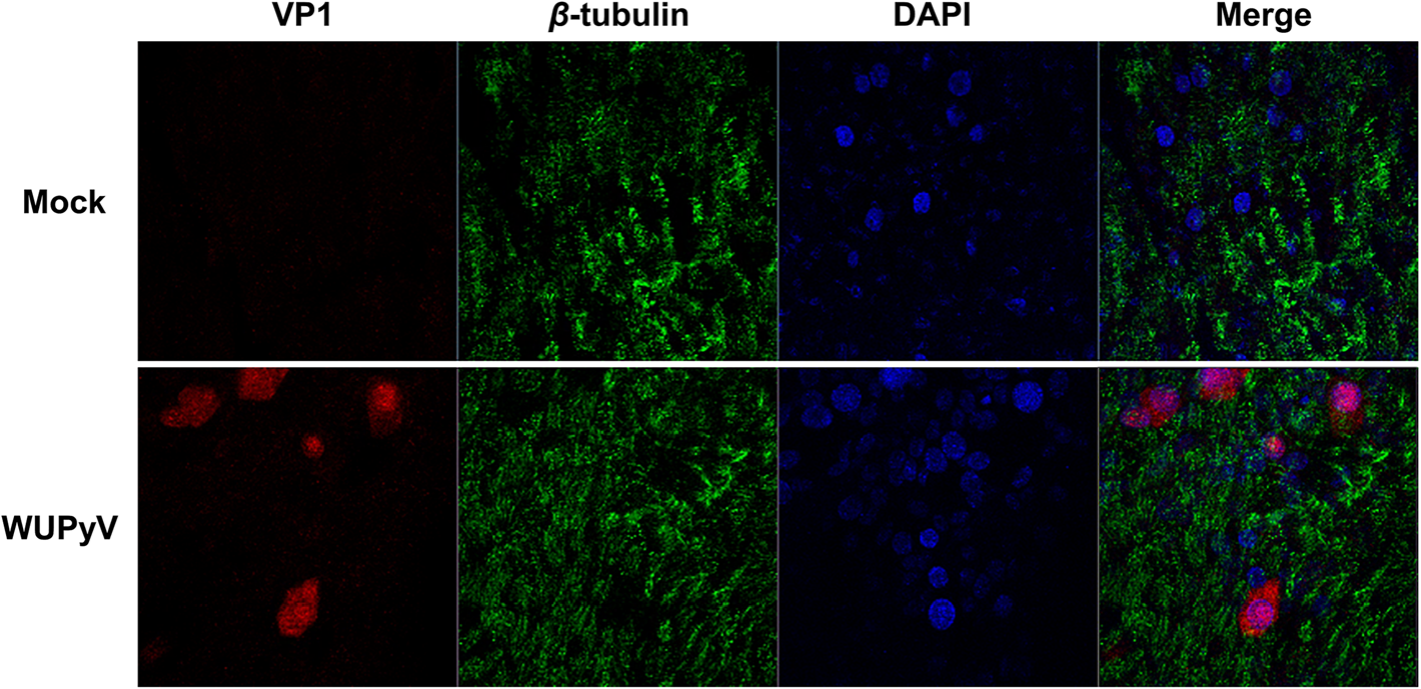
Immunofluorescence of WUPyV in HAE cells. HAE cells were infected with P1 progeny viruses, at 14 dpi (days post infection), then cells were co-stained with WUPyV-VP1 antiserum (red) and *β*-tubulin antibody (green). Nuclei were counterstained with DAPI (blue). Confocal images were obtained at a magnification of 60 ×

To confirm the infection of WUPyV in HAE cells, the harvested P1 progeny viruses were used to re-infect HAE cells. Both experimental and control groups were fixed with 4% paraformaldehyde at 14 dpi, and examined by immunofluorescence. The newly generated rabbit antiserum was used to detect WUPyV-VP1 protein, and ciliated cells were probed with a specific mouse monoclonal antibody, *β*-tublin [13, 16]. We detected *β*-tublin-specific fluorescence in both the experimental and control groups, indicating that HAE cells had differentiated into ciliated cells. In WUPyV-infected HAE cells, VP1-specific fluorescence was observed in both the cytoplasm and nucleus, with the nucleus appearing enlarged and round in shape. By contrast, no immunoreactivity for VP1 was detected in mock cells, and their nuclei showed no morphological changes (Fig. 2).

### Electron microscopy of WUPyV

**Fig. 3:**
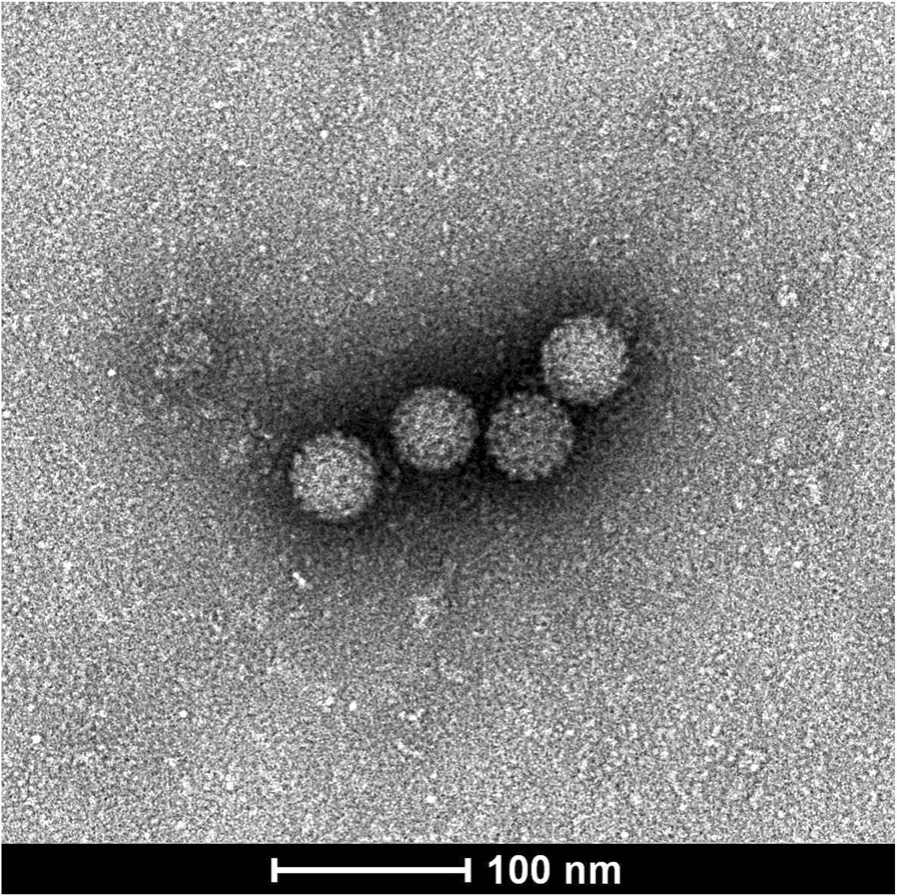
Transmission electron micrographs of WUPyV. WUPyV virions from the supernatant were negatively stained and examined by transmission electron microscopy. Scale bar represents 100 nm

To determine whether HAE cells could release complete virions, cells were re-infected with P1 progeny viruses. When the viral genome copy number reached 10^9^ copies/μL, the supernatant was collected and then observed by electron microscopy. All of the virions appeared as regular spherical particles and displayed the typical morphology of polyomaviruses, with a non-enveloped icosahedral structure and a diameter that varied from ~ 45 to 50 nm (Fig. 3).

### Whole genome analysis of WUPyV

**Fig. 4:**
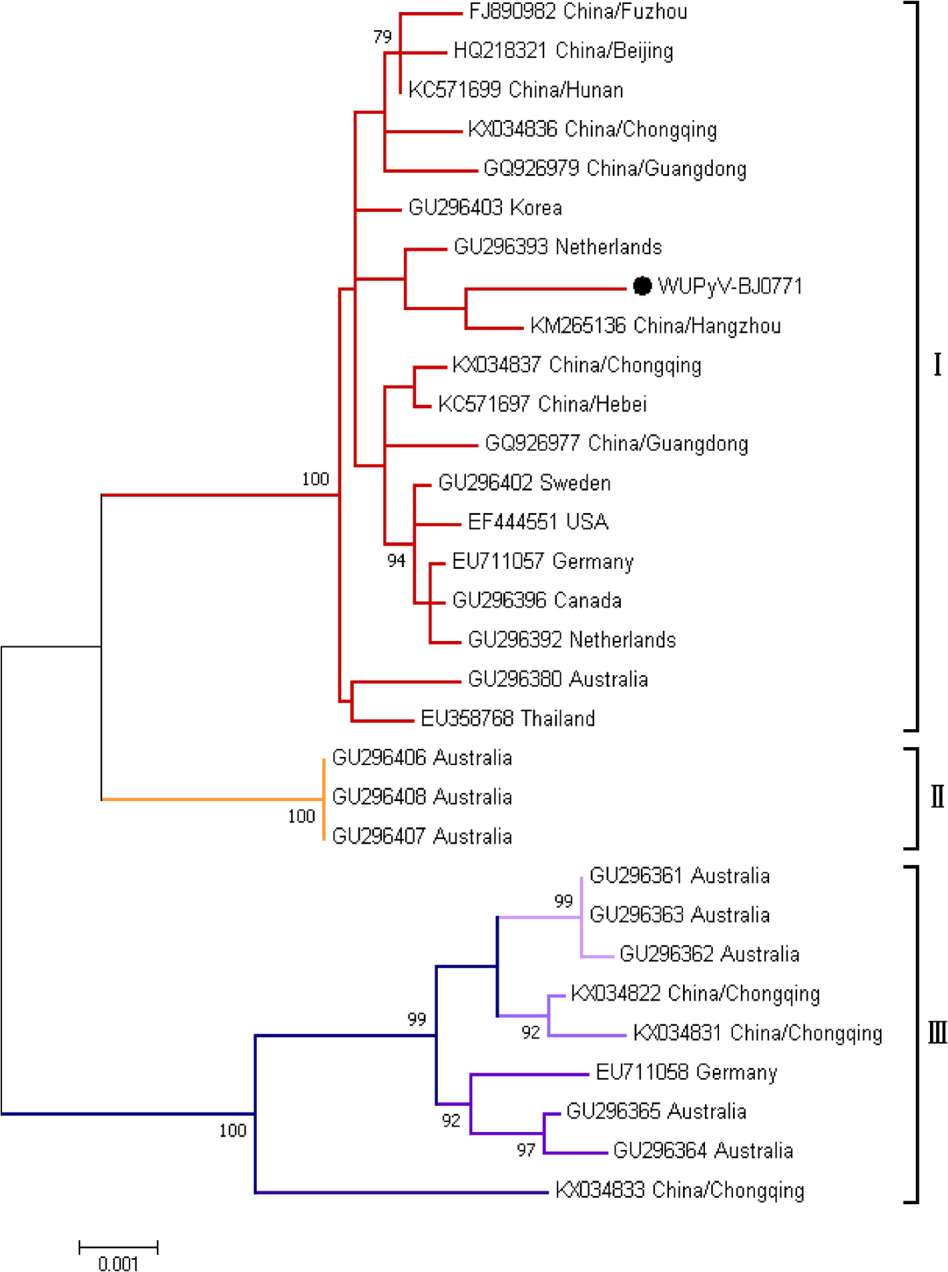
Phylogenetic tree of WUPyV. The maximum likelihood tree was constructed based on the complete genome sequence of the WUPyV clinical isolate using MEGA 7.0 software. Thirty additional sequences were included as reference strains. Branch points were labeled with bootstrap values, and the strain analyzed in this study was marked with a solid black circle

To characterize the WUPyV strain (BJ0771), the complete genome was sequenced using six pairs of primers with a partial overlap. The WUPyV genome is a circular double-stranded DNA of 5229 nucleotides (nt) in length, composed of two early regions encoding the large and small tumor antigens (LT and ST), and three late regions encoding the structural proteins (VP1, VP2, and VP3). Thirty WUPyV reference genome sequences were downloaded from the GenBank database for phylogenetic analysis. A phylogenetic tree was constructed using the maximum likelihood method and MEGA 7.0 software. WUPyV strains could be divided into three main clusters (I–III). Strain BJ0771 belonged to cluster I, which was closest to the WUPyV**-**Hangzhou (China) strain in genetic distance. The sequence of BJ0771 did not show any significant deviations, with ~ 99.3% and ~ 99.0% identity at the nucleotide and amino acid levels, respectively, compared with the selected reference strains. The whole genome sequence of BJ0771 was deposited in the GenBank database under accession no. MT066199 (Fig. 4).

## Discussion

The polyomavirus family contains four genera, *Alpha*-, *Beta*-, *Gamma*-, and *Deltapolyomavirus*. To date, a total of 102 polyomaviruses have been reported around the world, 13 of which are human polyomaviruses (HPyVs), including BKPyV and JCPyV identified in the early 1970s, and 11 types of HPyV detected since 2007, including KIPyV, WUPyV, and MCPyV [2]. Additionally, Quebec polyomavirus (QPyV) discovered recently from human fecal samples through MinHash algorithm has not yet be included by ICTV [22]. HPyVs can cause diseases of the nervous system, hematopoietic system, urogenital tract, and skin [6], with two main characteristics. First, polyomavirus infections are usually persistent but asymptomatic, only leading to illness in immunosuppressed and immunocompromised cases, such as TSPyV causing skin disease trichodysplasia spinulosa (TS) in severely immunocompromised patients [23]. Second, some virus-encoded oncogenic proteins can induce cell transformation and malignancies, such as MCPyV acting as a major causal factor in the development of Merkel cell carcinoma (MCC) [24]. WUPyV has been widely detected from respiratory tract samples around the world since it was first identified in 2007. WUPyV was usually asymptomatic or only caused mild symptomatic infections, and was often co-detected with other common respiratory viruses, with co-infection rates ranging from 26.7 to 100.0% [4]. However, WUPyV was the only pathogen detected in some patients [3, 25], prompting the study of its infectivity and pathogenicity. Since WUPyV is difficult to isolate in vitro, establishing a suitable culture system is especially important.

To date, there have been no cell lines available for the proliferation of HPyVs from clinical samples, with the exception of JCPyV and BKPyV [26]. Much of the research on polyomaviruses was performed using recombinant virions and pseudoviruses [27, 28]. Previous studies showed that WUPyV-VP1 could be detected in cytokeratin^+^ cells (epithelial cells), CD68^+^ cells (macrophage/monocyte lineage), and MUC5AC^+^ cells (goblet cells) in the lung tissue by immunohistochemistry [11, 29], indicating that WUPyV has obvious tropism for respiratory epithelia cells. Reconstructed HAE cells can develop a pseudostratified structure, and differentiate into basal cells, ciliated cells, and goblet cells, which could realistically simulate the virus infection environment in vivo, and was therefore proposed for use in the isolation of WUPyV derived from respiratory samples.

In this study, ALI-HAE cells cultured on Transwell plates were used for clinical sample inoculation, and a WUPyV strain (BJ0771) was successfully isolated from a 3-year-old child with respiratory infection. The replication curves showed that WUPyV grew rapidly in the early stage, with viral loads reaching a plateau at 6 dpi. Despite the growth rate slowing after day 6, viral loads remained at a constant high level (~ 10^9^ copies/μL), indicating that WUPyV could cause a persistent infection. Viral nucleic acids were detected in the basolateral chamber, suggesting that WUPyV could break through the respiratory epithelial barrier, being released into the basal medium from the surface of pseudostratified cells. The ability to break through this cell barrier is crucial for virus release and disease development, as the progeny viruses then have the opportunity to infect other cells with the same receptors.

HAE cells were re-infected with the harvested viruses, and immunofluorescence revealed that WUPyV could express the late region-encoded protein (VP1) in the HAE cell system. Electron microscopy provided evidence that complete WUPyV virions could be released. These two experimental techniques confirmed that HAE cells could produce infectious WUPyV.

Moens et al. [30] explored the activity of early and late promoters of HPyVs in 10 cell lines and the results showed that WUPyV has both higher early and late promoter activity on A375 cells, suggesting that the A375 cell line may be suitable for WUPyV isolation. We cultured the harvested viruses in A375 cells and four other traditional cell lines (293 T, Hep2, LLC-MK2, and MDCK); however, nucleic acid tests did not show significant proliferation (data not shown).

At present, international classification standards for WUPyV have not been published. Previous studies indicated that WUPyV could be divided into three main clusters (I–III) and several sub-clusters (Ia–Id and III a–IIIc) [4, 31], but the topological structure of the phylogenetic tree shows inconsistencies according to different algorithms [2]. In this study, we observed three clusters in the maximum likelihood tree, but no sub-groups were evident in cluster I. BJ0771 strain belonged to gene cluster I and was most closely related to a strain isolated in Hangzhou, China. Homology analysis showed that the genome of BJ0771 was highly conserved, at both the nucleotide and amino acid level, with other isolated strains.

## Conclusions

In summary, using the ALI-HAE cell system, WUPyV strain BJ0771 was successfully isolated in vitro for the first time in the world, and the replication kinetics and morphological characteristics of the virus were clarified. Additionally, phylogenetic analysis of its whole genome showed that BJ0771 belongs to cluster I. In future research, we will focus on the cellular receptors, cell tropism, and pathogenicity of WUPyV, and further explore the possibility of WUPyV culture on passaged cell lines.

## Data Availability

The datasets used and/or analyzed during the current study are available from the corresponding author on reasonable request.
